# A New Perspective for Bone Tissue Engineering: Human Mesenchymal Stromal Cells Well-Survive Cryopreservation on β-TCP Scaffold and Show Increased Ability for Osteogenic Differentiation

**DOI:** 10.3390/ijms23031425

**Published:** 2022-01-26

**Authors:** Liudmila Leppik, Anna Gempp, Zyrafete Kuçi, Selim Kuçi, Peter Bader, Halvard Bönig, Ingo Marzi, Dirk Henrich

**Affiliations:** 1 Department of Trauma-, Hand- and Reconstructive Surgery, University Hospital Frankfurt, Goethe-University, 60590 Frankfurt am Main, Germany; gempp.anna@gmail.com (A.G.); Ingo.Marzi@kgu.de (I.M.); d.henrich@trauma.uni-frankfurt.de (D.H.); 2 Department for Children and Adolescents, Division for Stem Cell Transplantation and Immunology, University Hospital Frankfurt, 60590 Frankfurt am Main, Germany; Zyrafete.Kuci@kgu.de (Z.K.); Selim.Kuci@kgu.de (S.K.); Peter.Bader@kgu.de (P.B.); 3 Institute for Transfusion Medicine and Immunohematology, Goethe University, German Red Cross Blood Service BaWüHe, 60528 Frankfurt am Main, Germany; Halvard.Boenig@kgu.de

**Keywords:** human MSCs, cryopreservation, osteogenic differentiation, scaffold, 3D culture

## Abstract

The clinical breakthrough of bone tissue engineering (BTE) depends on the ability to provide patients routinely with BTE products of consistent pharmacological quality. The bottleneck of this approach is the availability of stem cells. To avoid this, we suggest immobilization of random-donor-derived heterologous osteoinductive MSCs onto osteoconductive matrices. Such BTE products could then be frozen and, after thawing, could be released as ready-to-use products for permanent implantation during surgery. For this purpose, we developed a simple protocol for cryopreservation of BTE constructs and evaluated the effects of this procedure on human MSC (hMSCs) metabolic and osteogenic activity in vitro. Our findings show that hMSCs can be freeze-thawed on a β-TCP scaffold through a technically simple procedure. Treated cells sustained their metabolic activity and showed favorable osteogenic potential. Mechanistically, HIF1α and YBX1 genes were activated after freeze-thawing, and supposed to be linked to enhanced osteogenesis. However, the detailed mechanisms as to how the cryopreservation procedure beneficially affects the osteogenic potential of hMSCs remains to be evaluated. Additionally, we demonstrated that our BTE products could be stored for 3 days on dry ice; this could facilitate the supply chain management of cryopreserved BTE constructs from the site of manufacture to the operating room.

## 1. Introduction

Large non-healing bone defects, largely irrespective their etiology, constitute a major challenge for patients and physicians [1,2,3]. Treatment of choice is with autologous bone grafts [4]; however, several other treatments are also used [5,6,7]. These methods are not always effective, but even where they are, the morbidity associated with autologous tissue harvesting is considerable, fueling the search for alternatives.

Bone tissue engineering (BTE) is one such alternative approach, which holds great promise for promoting bone healing and regeneration while overcoming some of the drawbacks of current techniques. BTE approaches typically combine bone forming stem or progenitor cells with scaffolds, which restore missing bone volume and signaling molecules, which control cell–cell and cell–scaffold interactions in the bone defect. Among many different materials used to construct scaffolds, the biodegradable ceramic scaffolds with desirable pore size, porosity, and mechanical properties are probably the most preferable in BTE, since these materials generally show better tissue responses compared to polymers and metals [8]. Tricalcium phosphate (TCP) ceramics in contrast to other ceramics (for example hydroxyapatite) found wide application in the clinic due to their osteoconductivity and bioresorbability [9]. Clinical BTE approaches have demonstrated encouraging early outcomes [10]. However, in these types of approaches availability of stem cells is a bottleneck of the whole procedure. Isolation, characterization, and expanding of autologous mesenchymal stem cells (MSCs) or use of cryopreserved dissociated stem cell populations from cryobanks [11] need significant investment of time and resources that limits translation of BTE approaches into clinic. To be able to provide BTE products of consistent medicinal quality, conducive to routine clinical use, we proposed to immobilize random-donor-derived osteoinductive MSCs onto osteoconductive matrices. These BTE products were then frozen and released ready-to-use for permanent implantation during surgery after thawing.

The work builds on earlier work which has definitively established that cryopreserved MSCs maintain potential for proliferation and osteogenic differentiation in vitro [12,13,14,15] and in vivo [16,17]. The methods used, however, were not conducive to clinical application. A recent pilot study of Hernandez–Tapia showed that osteoblasts have good survival rate after cryopreservation on β-TCP scaffold [18]. The equally recent study of Mutsenko et al. [19] showed that MSCs could be frozen in 3D collagen-hydroxyapatite scaffold without significant loss of cell viability. Although these studies use technically challenging methodology, they represent invaluable first steps towards the creation of a tissue-engineered constructs biobanks.

In the present study, we developed a simple protocol for cryopreservation of BTE constructs (human MSCs seeded on 3D β-TCP scaffold) and evaluated the effects this procedure has on human MSCs metabolic and osteogenic activity in vitro.

## 2. Results

### 2.1. Effect of Cryopreservation on hMSCs Metabolic Activity

**Figure 1:**
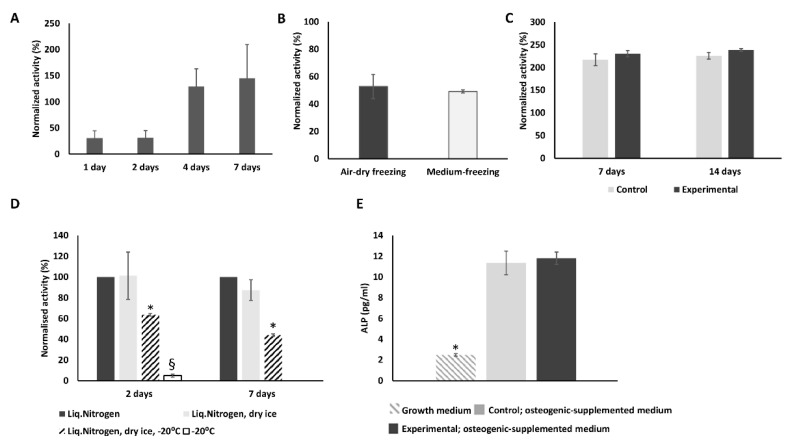
hMSCs preseeded on scaffolds retain their metabolic activity after cryopreservation. (**A**) Metabolic activity of MSCs was measured 1, 2, 4, and 7 days after cryopreservation, shown in % of control cells metabolic activity. MSCs were preseeded on scaffolds 48 h prior to cryopreservation. (**B**) There was no significant difference in metabolic activity of hMSCs processed with two different cryopreservation protocols and measured 2 days after cryopreservation. (**C**) Metabolic activity of control and experimental cells at 7 and 14 days of culture in osteogenic supplemented medium showed no significant differences. (**D**) hMSCs were first cryopreserved in liquid nitrogen and either stored for 3 days on dry ice or stored for 3 days on dry ice and 1 day at −20 °C. The metabolic activity of cells was measured at days 2 and 7 post-thaw and is shown in % of metabolic activity of cells stored in liquid nitrogen only. (**E**) ALP expression is significantly enhanced in both, control and experimental group cells at day 7 of culture in osteogenic-supplemented medium, as compared to cells cultured in growth medium. (* *p* < 0.05, ^§^ *p* < 0.01).

First, we analyzed how cryopreservation affects the metabolic activity of cells frozen on scaffold granules following common freezing protocol and whether the cells could recover their activity with time. Our results show that MSCs retained approximately 40% of their metabolic activity at first two days after cell thawing as compared to control (not frozen) cells (Figure 1A), and at later time points cells increased their activity to the level of control cells. We also compared recovery of cells frozen in this way with the cells frozen by mean of “air-dry” freezing protocol. No significant difference in metabolic activity of these two groups of cells post-thaw was detected for all time points (Figure 1B). Based on this the “air-dry” protocol was chosen for the following experiments.

In addition, we analyzed if “air-dry” 3D cryopreserved hMSCs could be stored at −20 °C, in dry ice or in a combination of both. We compared the metabolic activity of cryopreserved cells stored in (1) liquid nitrogen, (2) liquid nitrogen and −20 °C, (3) liquid nitrogen and dry ice (3 days), and (4) liquid nitrogen, dry ice (3 days) and −20 °C (1 day) after thawing. Our results show that cells did not survive storage at −20 °C (Figure 1D); however, storage in dry ice for 3 days did not negatively affect cell metabolic activity after thawing. The combination of both storage conditions, dry ice, and −20 °C had a significant negative effect on cell metabolic activity at 2 and 7 days after thawing (Figure 1D).

**Figure 2:**
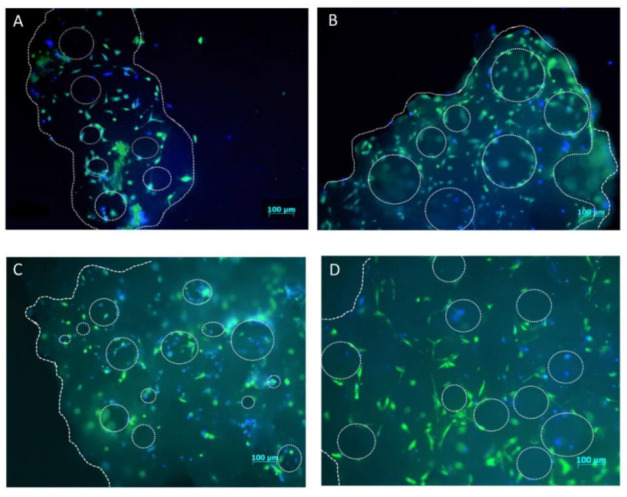
hMSCs distribution on a β-TCP scaffold granule before and post-thawing. Control hMSCs were seeded on a β-TCP scaffold and cultured either for 24 (**A**) or 72 h (**B**). Experimental hMSCs were seeded on a β-TCP scaffold, cultured for 24 h, frozen in liquid nitrogen for seven days, thawed, and cultured for 1 (**C**) and 48 (**D**) hours. All cells are stained with Calcein AM and DAPI. Dotted line shows the edges and pores of β-TCP granule. (5× magnification; scale bar = 100 µM).

To compare the cell distribution on a scaffold before and after freezing, live cells were visualized with Calcein AM and DAPI staining. As can be seen from Figure 2, there were less cells present on a granule surface and more cells present inside pores in the samples 1-h post-thaw (Figure 2C,D) as compared to control samples without freezing.

### 2.2. Cryopreserved hMSCs Show Stronger Osteogenic Activity Than Control Cells

In order to analyze if cryopreservation has effect on osteogenic potential of hMCS post-thawing and therefore on future use of these cells in BTE approaches, cells after cryopreservation were thawed and cultured for two weeks in osteogenic-supplemented medium (Figure 1C). There was no difference in the level of alkaline phosphatase (ALP) expression among control and cryopreserved cells at day 7 of culture in osteogenic-supplemented medium. In both type of cells expression of ALP was significantly higher than in cells cultured in growth medium (Figure 1E).

**Figure 3:**
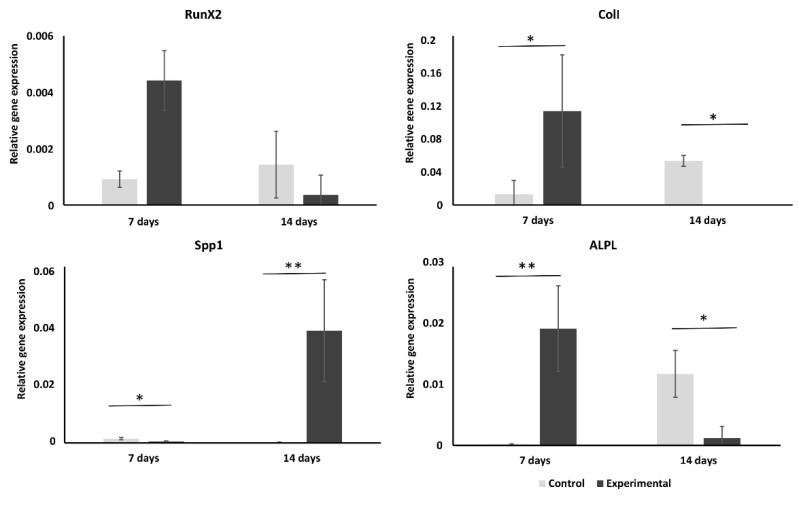
Osteogenic marker genes mRNAs expression in control and experimental cells. Expression of osteogenic marker genes was evaluated in experimental and control group hMSCs at days 7 and 14 of culture in osteogenic-supplemented medium. (* *p* < 0.05; ** *p* < 0.01).

The results of osteogenic gene expression analysis are presented in Figure 3. Surprisingly, the expression of all tested genes was enhanced in the experimental group as compared to control (not frozen) cells. Expression of early markers of osteogenesis RunX2, Col1, and ALPL in the experimental group peaked on day 7 of osteogenic culture, whereas in the control group it did not peak until day 14. Col1 expression was significantly higher (*p* < 0.05) in experimental group cells as compared to control cells at day 7 of osteogenic culture. On the contrary, its expression was significantly (*p* < 0.05) higher in control group cells at day 14 of culture. Expression of the SPP1 gene was low in both groups of cells on day 7, but was significantly increased on day 14 in the experimental group as compared to control group cells (*p* < 0.01). None of osteogenic marker genes was expressed in cells cultured in growth medium except for SPP1, which showed strong up regulation on day 14 of culture (Supplementary Figure S1). In the control-DMSO group, in which cells were treated with freezing medium but were not frozen, expression of osteogenic marker genes was similar as in control cells except for Col1. This gene was highly upregulated in these cells at day 7 of osteogenic culture (Supplementary Figure S1).

### 2.3. Cryopreservation Has an Effect on Expression of Hypoxia-Related Genes

**Figure 4:**
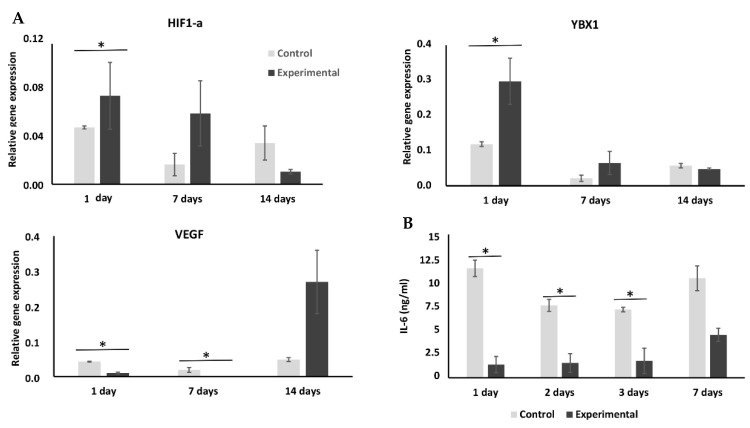
mRNA levels of gene expression of HIF1-α, YBX1, and VEGF and IL-6 protein secretion at days 1, 7, and 14 of culture. (**A**) Expression of HIF1α, YBX1, and VEGF genes was evaluated in experimental and control group hMSCs at days 1, 7, and 14 of culture in osteogenic-supplemented medium. (**B**) Concentration of IL-6 was measured in medium supernatants of both groups at days 1, 2, 3, and 7 of culture. (* *p* < 0.05).

Hypothesizing that the positive effect of cryopreservation on hMSCs osteogenic differentiation could be promoted by the difference of oxygen concentration developed due to temperature difference, we compared expression of hypoxia-related genes in experimental and control cells (Figure 4). The results showed that expression of the major hypoxia marker gene HIF1α and several of its target genes was affected by cryopreservation (Figure 4 and Supplementary Figure S2). Expression of HIF1α in the experimental group cells was significantly (*p* < 0.05) higher than in the control cells one day post-thaw. On day seven, expression of HIF1α and its target genes PDK1, SLC2A1, EGLN1, and BNIP3 was higher in cells which had undergone the cryopreservation procedure compared to control cells, albeit not significantly. Expression of VEGF mRNA was significantly lower on days 1 and 7, and significantly higher on day 14 of culture in the experimental group cells as compared to controls (Figure 4A).

### 2.4. Cryopreservation Enhances YBX1 Expression but Has No Effect on Wnt/Smad Pathways

We analyzed if the freezing procedure has an effect on expression of the cold shock protein YBX1, known to react to low temperature and to be an important regulator of transcription [20]. Expression of this gene was significantly (*p* < 0.05) enhanced as early as 1 day after thawing in the experimental group and remained high until day 7 of culture. On day 14 of culture, expression of this gene was the same in both experimental and control group cells (Figure 4).

In order to investigate if the freezing procedure could have effect on key signaling (Wnt and SMAD) pathways in hMSCs and thereby influence hMSCs osteogenic potential, we analyzed expression of Wnt3a, SMAD5, MAPK8, and MAPK14 genes early after the freezing and thawing event (1 day) as well as at later time points (7 and 14 days). No significant difference in expression of these genes among experimental and control group cells was found at any time point (Supplementary Figure S3).

### 2.5. Effect of Cryopreservation on Interleukin Expression

We evaluated if cryopreservation has an effect on hMSC’s IL-10 and IL-6 expression 1–7 days into osteogenic culture, as the mediators could be important for future use of these cells in BTE approaches (Figure 4B). In both control and experimental cells, expression of IL-10 was below detection level of our method (data not shown). Concentration of IL-6 was lower in the experimental group on days 1, 2, and 3 as compared to control-group medium supernatant. On day 7 of osteogenic culture, the difference in expression of IL-6 between two groups of cells lost statistical significance.

## 3. Discussion

The clinical breakthrough of bone tissue engineering depends on the ability routinely to provide patients with BTE products of consistent pharmacological quality. For this reason, the ability to batch-produce and cryogenically preserve tissue engineered construct represents major progress. Cryopreservation is the use of very low temperature to maintain living cells and tissues in a quiescent status for a long period, without losing their structure and function [21]. Several works have been devoted to the cryopreservation of engineered biological constructs [18,19,22,23,24], which could guide our experimentation on the cryopreservation of biofabricated BTE constructs.

In the present study we evaluated whether hMSCs retain their activity after cryopreservation on β-TCP scaffolds. Our results showed that despite the blunted metabolic activity at early time points after thaw and presumably representing a modicum of cell attrition, hMSCs quantitatively regain their metabolic activity later. This temporal loss of metabolic activity could be explained by the loss of cells from the scaffold granule surface, as was shown by Calcein and DAPI staining of cells one-hour post-thaw (Figure 2C, Supplementary Figure S4). These findings are in accordance with other studies, showing that *Callithrix jacchus* MSCs and cells of human osteoblastic cell lines sustain their activity after being frozen on hydroxyapatite or β-TCP scaffolds, respectively [18,19]. In contrast to both these studies, we employed the simplest cell culture and cryopreservation procedures in order to develop protocols which can be later easily translated to clinical application. Therefore, we used a commonly used β-TCP scaffold, MSCs generated from pooled bone marrow mononuclear cells of eight third-party donors and an “air-dry” cryopreservation protocol, which involved the use of standard cell culture equipment and reagents. We also evaluated whether 3D-cryopreserved hMSCs could survive storage at −20 °C, dry ice, or a combination of both. Our results clearly demonstrate that cells seeded on β-TCP scaffold and stepwise cryopreserved could not be stored at −20 °C as that dramatically effects metabolic activity of cells post-thaw. However, cryopreserved BTE constructs could be stored at least for 3 days on dry ice without an apparent impact on post-thaw cell activity. Cryopreserved hMSCs-coated matrix also survived the storage on dry ice followed by 1 day at −20 °C, albeit with significant loss of metabolic activity post-thaw. This information will guide the supply chain management of cryopreserved BTE constructs from the site of manufacture to the operating room.

Osteogenic potential is arguably one of the most important characteristics of biofabricated BTE constructs; therefore, we evaluated how cryopreservation on 3D scaffold affects hMSCs osteogenic potential. Our results showed that osteogenic potential of frozen cells was rather improved. ALP activity of control and experimental cells was the same at day 7 of osteogenic culture (Figure 3). According to the osteogenic marker gene expression analysis, hMSCs osteogenic differentiation was accelerated after freezing/thawing procedure, as most of the analyzed markers were expressed earlier in experimental cells than in control cells. It has been previously shown that MSC retain osteogenic differentiation ability after cryopreservation [25,26], but for hMCSs cultured and cryopreserved on β-TCP scaffold this was not shown before. The effect of increased osteogenic potential was not observed in our “DMSO-control” cells, demonstrating that increased osteogenic gene expression is rather induced by physical effects and not by components of the cryopreservation medium (e.g., DMSO).

We hypothesized that increased osteogenic gene expression could either be a result of unspecific up-regulation of major pathways due to the cryopreservation procedure and/or reaction to the oxygen gradient induced by temperature changes [27,28]. To validate this hypothesis, we analyzed expression of key signaling stem cell fate marker genes and expression of hypoxia-related genes. Results of this analysis showed that expression of Wnt3A, MAPK8, MAPK14, and SMAD5 was not significantly altered in cryopreserved cells (Supplementary Figure S1), whereas the expression pattern of hypoxia related genes differed between control and experimental cells. Expression of HIF1α gene and its target genes PDK1, SLC2A1, EGLN1, and BNIP3 was upregulated in experimental group cells on day 7, whereas in control cells those genes were only upregulated on day 14 of culture. It was previously shown that hypoxia promotes osteogenesis of human MSCs in a HIF-1-dependent manner [29]. We speculate that the cryopreservation procedure affects HIF1α expression, and this mechanism stimulates osteogenesis. In addition, VEGF, a known target of HIF1α, was also significantly upregulated in experimental group cells (Figure 4). A previous study had shown that RunX2 acts together with HIF1α to stimulate angiogenic gene expression in bone cells [30]. Our results are in accordance with it.

We also verified if transcription of cold shock protein YBX1, known to regulate expression of HIF1α [31], was activated after cryopreservation (Figure 4). YBX1 protein possess a cold shock domain and stimulates translation of proteins under low temperature [20,32]. YBX1 protein was shown to be a critical regulator of HIF1α expression in sarcoma cells [33]; however, less is known about the role of this protein in MSCs. Our results show that cryopreservation affects YBX1 gene expression in hMSCs, which would be compatible with a role in the response of the MSCs to cryopreservation and enhancement of their osteogenic potential.

## 4. Materials and Methods

4.1. hMSCs Culture and Seeding on β-TCP Granules

Human bone marrow MSC cells (hMSCs) were provided by Department for Children and Adolescents, Division for Stem Cell Transplantation and Immunology, University Hospital Frankfurt. The MSCs were generated from pooled, previously isolated, and cryopreserved mononuclear cells from eight random bone marrow donors by plastic adherence, which have been expanded to near-confluence and cryopreserved in small aliquots as described in detail elsewhere [34]. It has already been demonstrated that these MSCs were effective in the treatment of patients with acute steroid-resistant acute GvHD [35,36]. For subcultures, 3.5 × 105 hMSCs were seeded in T175 cell culture flasks (Sarstedt, Nümbrecht, Germany) and cultured in animal-free growth medium (10% CruxRufa Media Supplement; (TrinovaBiochem, Giessen, Germany); 1 IU/mL of heparin (Ratiopharm, Ulm, Germany); GlutaMax Gibco™ DMEM, (Thermofischer, Dreieich, Germany)) at 37 °C, 5% CO_2_ in a humidified incubator until 80–90% confluence. The culture medium was changed every three to four days and the cells were expanded until passage 5. These MSCs were used either for experiments or stored in liquid nitrogen for future use. Before cryopreservation, hMSC was assessed for expression of typical MSC cell surface markers by using flow cytometry analysis. Phenotypical analysis showed that these cells express high levels of surface proteins CD90 and CD105, but very low or no expression of hematopoietic cell markers CD45 and CD34 (data not shown [34,37]).

For 3D hMSC culture, 500 µL of β-TCP scaffold granules (ChronOS Granules, 1.4–2.8 mm, 60% porosity; Synthes, Oberdorf, Switzerland) were placed in 6.5-mm Transwell ^®^ membrane inserts (3.0 µm pore, polycarbonate, Corning, Wiesbaden, Germany) allocated in individual wells of a 24-well plate. Then 2 × 10^5^ hMSCs in 150 µL of PBS (Gibco) were dripped slowly on the scaffolds and incubated for 10 min at 37 °C. After incubation, the cell suspension not absorbed by the scaffold was removed, dripped again over the material, and incubated for another 10 min. This step was repeated twice. Afterwards, 1 mL of pre-warmed growth medium was added to each well and cells were further incubated at 37 °C, 5% CO_2_ in a humidified incubator for 24 h.

### 4.2. Effect of Cryopreservation on Cell Activity

In order to evaluate the effect of cryopreservation on cell metabolic activity, osteogenic differentiation, and interleukin expression, the scaffold granules with seeded cells were frozen, thawed, and cultured for two weeks in either growth or osteogenic-supplemented medium (experimental group). In the control group, cells were treated the same way as in experimental group except for freeze-thawing.

### 4.3. Cryopreservation and Thawing of hMSCs on β-TCP-Scaffold

For “standard protocol” cryopreservation, scaffold granules seeded with cells and cultured for 24 h were transferred into a cryovial, and entire culture medium was exchanged with 1 mL of cold fresh-prepared freezing medium (10% *v*/*v* DMSO, 25% human AB serum (Sigma–Aldrich, Heidelberg, Germany) in DMEM Glutamax). The cryovial with cells was stored at −80 °C for 24 h in a controlled rate freezing container (Nalgene ^®^ Mr. Frosty, Merck, Darmstadt, Germany) and then transferred into a liquid nitrogen for at least 7 days’ storage. For thawing, a cryovial with hMSCs-seeded β-TCP scaffold granules was removed from the nitrogen tank, briefly warmed in a water bath (37 °C) for 1 min. The cryovial content was transferred into one well of a 24-well-plate and the entire freezing medium was aspirated. Cells were washed with 1 mL of fresh, pre-warmed growth medium, and cultured in 1 mL of growth medium (at 37 °C; 5% CO_2_) for 48 h at which time the cell metabolic activity was assayed.

For the “air-dry protocol” cryopreservation [19], scaffold granules seeded with cells were transferred into a cryovial and the entire culture medium was exchanged with 1 mL of cold fresh-prepared freezing medium. After 15 min incubation on ice, medium was soaked out and cryovial with cell-coated scaffold (without medium) was first frozen at 80 °C in a controlled rate freezing container for 24 h, and subsequently stored in liquid nitrogen for at least 7 days. For thawing, a cryovial with hMSCs-seeded β-TCP scaffold granules was removed from the nitrogen tank, briefly warmed in a water bath (37 °C) for 1 min. One mL of pre-warmed growth medium was quickly added and the complete cryovial content was transferred into one well of a 24-well-plate. Cells were incubated at 37 °C, 5% CO_2_ for 48 h before cell metabolic activity was assayed.

In order to control for possible effects of DMSO in freezing medium on cell activity after thaw, cells in “DMSO control” group were treated the same way as described in “air-dry protocol”, but the freezing step (−80 °C, 24 h, and liquid nitrogen storage) was omitted.

### 4.4. Effect of Storage Temperature

To analyze if cryostorage temperature has an effect on the functionalized β-TCP scaffold, cryovials with 3D cryopreserved cells were first stored in liquid nitrogen (7 days) and then either directly thawed and cultured or stored for 1 day at: −20 °C (freezer); for 3 days in dry ice (Styrofoam container filled with dry ice = −78.5 °C); or for 3 days in dry ice and 1 day at 20 °C. Thereafter, cells were thawed as described and cultured for 48 h or 7 days before metabolic activity of cells was measured.

### 4.5. Cell Metabolic Activity Measurements

Metabolic activity of cells cultured on scaffold granules was measured by means of alamarBlue assay (Bio-Rad, Germany) according to the manufacturer’s protocol 48 h after thaw in experimental or 72 h after seeding in control groups, respectively. Briefly, growth medium was soaked out and 400 µL of fresh growth medium and 40 µL of alamarBlue reagent were added to the cell-seeded scaffold granules. After 4 h of incubation at 37 °C, absorbance of conditioned medium was measured at 570 and 600 nm by means of Tecan Plate reader (Tecan, Crailsheim, Germany) and the percentage reduction of alamarBlue was calculated according to the manufacturer’s instructions. Three samples were analyzed for each group and the mean value and standard deviation were calculated.

### 4.6. hMSCs Distribution on Scaffold Granules

To visualize hMSCs, seeded on β-TCP scaffold, before and after freezing-thawing procedure, Calcein AM (BD Pharmingen, Heidelberg, Germany) and DAPI (Life Technologies, Darmstadt, Germany) staining were applied. Scaffold granules with cells were transferred into a well of 24-well plate and 1 mL of pre-warmed growth medium with 20 µM Calcein AM was added. Cells were incubated for 40 min at 37 °C; washed several times with PBS; and DAPI (1 μg/mL in PBS) was added. After 10 min of incubation, cells were washed several times with PBS. hMSCs adherence and distribution on a scaffold was then assessed by directly viewing the samples with fluorescence microscopy using a Zeiss Axioobserver Z1 (Zeiss, Göttingen, Germany).

### 4.7. Osteogenic Differentiation

In order to evaluate the effect of cryopreservation on hMSCs osteogenic differentiation, cells after thawing were cultured for 24 h in growth medium and then growth medium was supplemented with 10-7 M of dexamethasone, 10 mM of β-glycerophosphate, and 0.05 mM of ascorbic acid-2-phosphate (Osteogenic medium, OM), all obtained from Sigma-Aldrich (Heidelberg, Germany). Cells from the control group were cultured in growth medium for 48 h after seeding, whereupon medium was changed to osteogenic-supplemented medium. Cells from both groups were cultured for another two weeks in osteogenic-supplemented medium and medium was changed every 3–4 days.

### 4.8. Gene Expression Analysis

For gene expression analysis, total RNA from cells was isolated using RNeasy Mini Kit (Qiagen, Hilden, Germany) following the manufacturer’s instructions. Purity and quantity of RNA were measured using an Infinite 200PRO NanoQuant device (Tecan, München, Germany). DNase-treated RNA samples were reverse-transcribed using iScript Select cDNA Synthesis Kit (Bio-Rad, Feldkirchen, Germany) according to the manufacturer’s instructions. The RT-qPCR reaction was performed using the cDNA equivalent of 10 ng RNA and the RT2 SYBR Green qPCR Mastermix (Qiagen, Hilden, Germany). All samples were amplified in duplicates using a CFX96 Touch Real-Time PCR Detection System (BioRad, Feldkirchen, Germany) with human gene specific primers (RT2 qPCR Primer Assays, Qiagen, Hilden, Germany), and thermal profile of 1 cycle with 10 min of 95 °C, 40 cycles with 15 s of 95 °C, and 1 min of 60 °C followed by dissociation curve. GAPDH was used as reference gene in each experiment. A melting curve analysis was applied to ensure the specificity of the PCR procedure. Relative quantification of messenger RNA (mRNA) levels of the target genes was analyzed using the comparative CT (threshold cycle values) method (2^−ΔCt^) [38]. The results are presented as relative quantification (RQ), which is expression fold change compared to the reference gene. Three samples were analyzed for each group and mean value and standard deviation were calculated for further analysis.

### 4.9. Alkaline Phosphatase Expression Assay

As a marker of osteogenesis, ALP expression was assessed on day 7 of osteogenic culture according to the manufacturer’s protocol (Sensolyte pNPP Alkaline Phosphatase Detection Kit, Anaspec Inc., Köln, Germany). Granules with seeded cells were washed twice with 1x assay buffer and cells were lysed with lysis buffer (0.02% Triton X-100 in 1× assay buffer). Cell lysates were collected into fresh micro tubes and processed for ALP activity measurements. Absorbance was measured at 405 nm by means of Infinite 200PRO NanoQuant plate reader (Tecan, Crailsheim, Germany). The absolute ALP value for each sample was calculated against an alkaline phosphatase standard curve.

### 4.10. ELISA

The effect of cryopreservation on interleukin (IL)-6 and IL-10 expression in hMSCs was evaluated using ELISA. The concentration of interleukins was measured in cell conditioned medium collected at days 1, 2, 3, and 7 post-thawing by means of human IL-6 DuoSet ^®^ ELISA and human IL-10 DuoSet ^®^ ELISA (R&D Systems, Wiesbaden-Nordenstadt, Germany) in accordance with the manufacturer’s instructions. The absorbance was measured at 450 nm and cytokine concentrations were calculated based on calibration curve plotted with different standards concentrations by means of Magellan Software.

### 4.11. Statistical Analysis

All experiments were done in triplicates. The data are presented as mean  ±  SD and significance level was set at *p* < 0.05. Nonparametric Kruskal–Wallis test followed by Bonferroni-corrected multiple Conover–Iman post hoc analysis was consequently applied. A *p* < 0.05 indicated statistical significance. Statistics were calculated using the software Bias 11.12 (Epsilon-Verlag, Darmstadt, Germany).

## 5. Conclusions

Our findings show that hMSCs can be freeze-thawed on β-TCP scaffold through a technically simple procedure and thus provide a critical step towards the development of clinical-grade BTEs for functional bone augmentation. Cells treated in this fashion sustained their metabolic activity and showed favorable osteogenic potential after cryopreservation. The detailed mechanisms as to how the cryopreservation procedure beneficially affects the osteogenic potential of hMSCs, specifically the roles of YBX1 and HIF1α genes, should be investigated in future studies.

## Data Availability

Not applicable.

## Supplementary Materials

The following are available online at https://www.mdpi.com/article/10.3390/ijms23031425/s1.
