# Supplementary material for: A New Perspective for Bone Tissue Engineering: Human Mesenchymal Stromal Cells Well-Survive Cryopreservation on β-TCP Scaffold and Show Increased Ability for Osteogenic Differentiation
Source: Int J Mol Sci. 2022 Jan 26;23(3):1425. doi: 10.3390/ijms23031425 (PMC8835857; doi:10.3390/ijms23031425)
Supplement: Supplementary file 1 [file ijms-23-01425-s001.zip › ijms-1535224-supplementary.pdf]

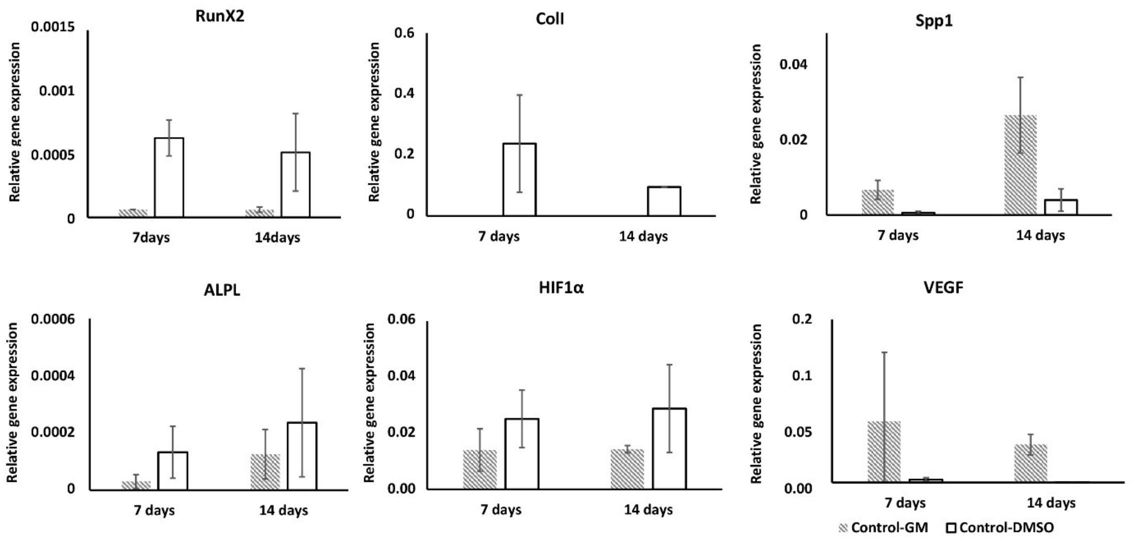

Supplementary figure S1. Osteogenic marker gene expression in hMSCs cultured in growth medium or DMSO-control group cells. Control –GM – hMSC were cultured for 7 or 14 days in growth medium. Control-DMSO – hMSCs were treated as experimental group cells, but the cryopreservation step was omitted.

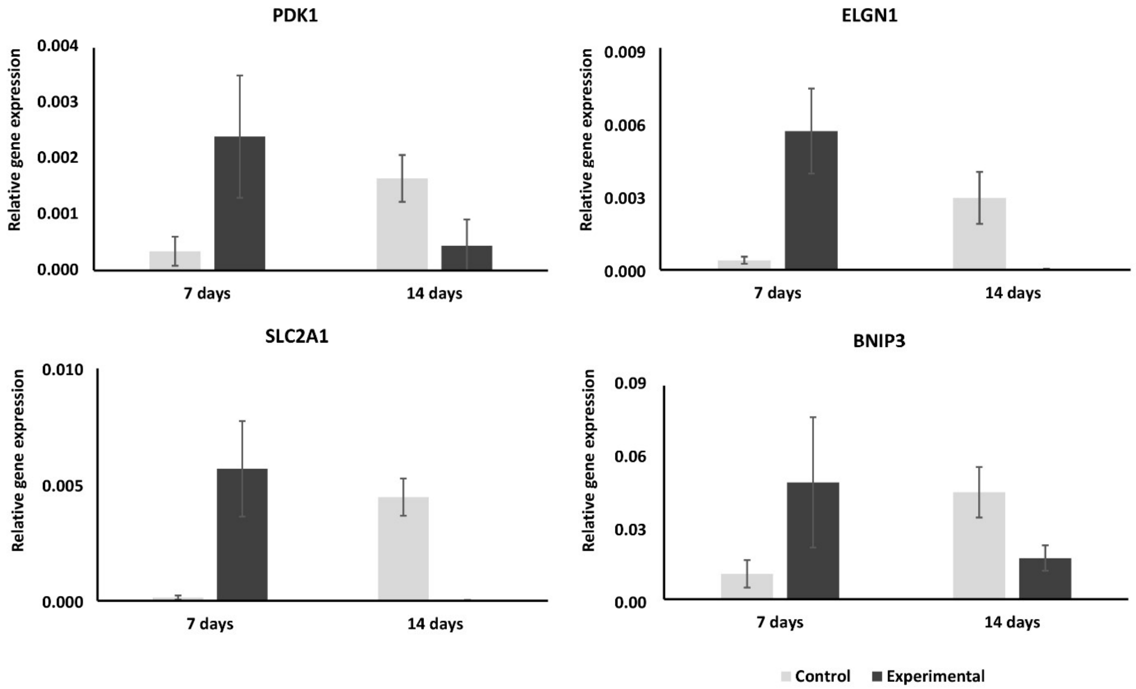

Supplementary figure S2. Expression of hypoxia related genes in experimental and control group cells. Expression of HIF1α-target genes in hMSCs was measured on days 7 and 14 of culture.

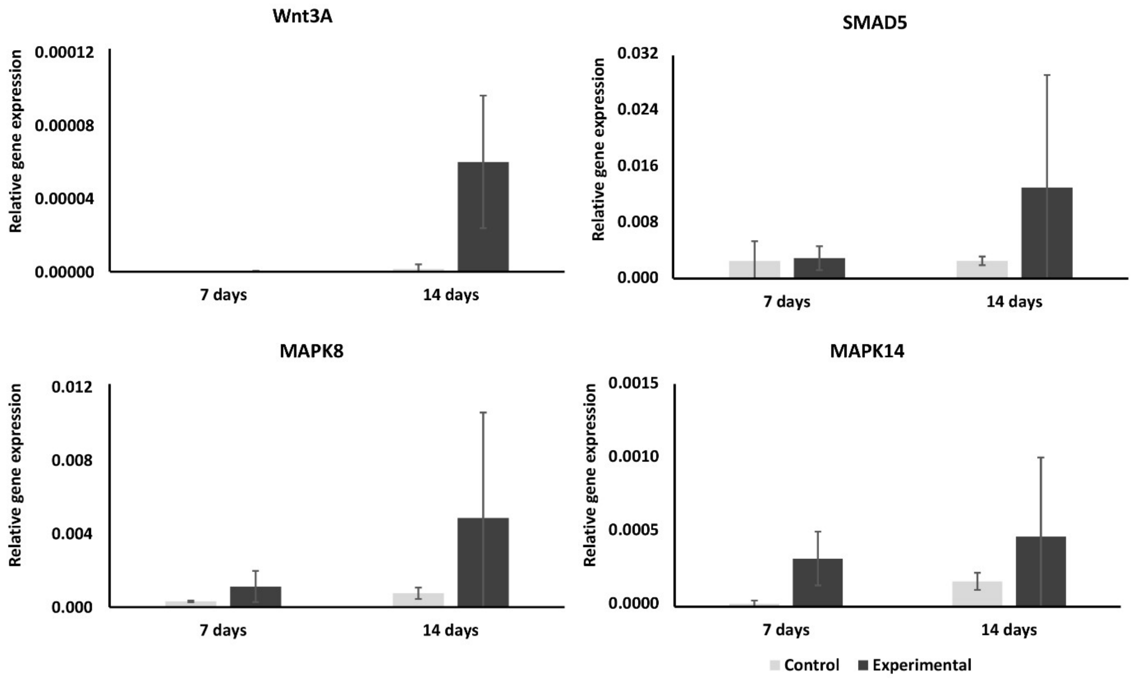

Supplementary figure S3. Expression of Wnt- and SMAD- pathway related genes in hMSCs. Expression of Wnt3A, SMAD5, MAPK8 and MAPK14 genes was measured in control and experimental group cells on days 7 and 14 of culture.

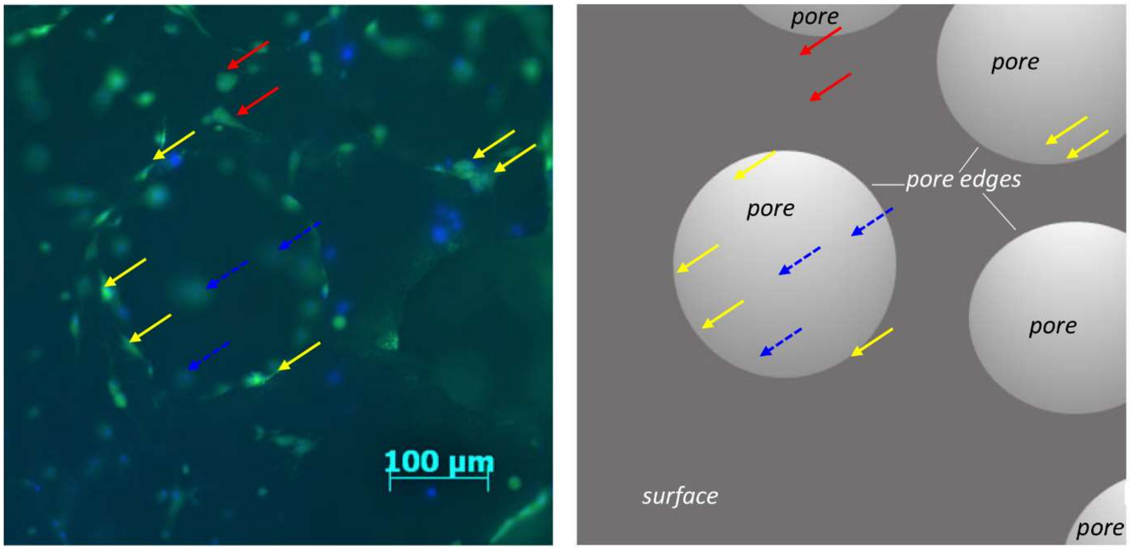

Supplementary figure 4. Representative high magnification image showing hMSCs distribution on a ß-TCP scaffold granule and schematic drawing showing scaffold structure. Control hMSCs were seeded on a ß-TCP scaffold and cultured for 24 hrs. Cells are stained with Calcein AM and DAPI. Red arrows show cells growing on the surface of the granule, yellow arrows show cells growing on the edge of the pore, dotted blue arrows show cells growing inside the pore of the granule. (10x magnification; Scale Bar = 100 μM)
